# Advancing cotton fiber research with variable-pressure scanning electron microscopy

**DOI:** 10.3389/fpls.2025.1562682

**Published:** 2025-05-01

**Authors:** Fang Bai, M. Andrew Jansen

**Affiliations:** ^1^ The United States Department of Agriculture (USDA), Agricultural Research Service, Crop Genetics Research Unit, Stoneville, MS, United States; ^2^ The United States Department of Agriculture (USDA), Agricultural Research Service, Beltsville Agricultural Research Center, Systematic Entomology Laboratory, Electron and Confocal Microscopy Unit, Beltsville, MD, United States

**Keywords:** variable-pressure scanning electron microscope (VP-SEM), scanning electron microscope (SEM), microscopy, cotton fiber, fiber initiation, fiber elongation

## Abstract

Cotton fibers, as highly extended, thickened epidermal seed structures, are a crucial renewable resource in textile production. Cotton plants produce two main types of fiber cells: wide, hemisphere-shaped fibers and narrow, tapered fibers. Both types stabilize through secondary cell wall development, with the mature narrow fibers being particularly valued for spinning into fine, strong yarns, suitable for premium cotton fabrics. Traditional methods for studying fiber development and cell types, such as scanning electron microscopy (SEM), are often time-intensive and costly. SEM preparation steps, including fixation, dehydration, and sputter coating, can cause shrinkage and other image distortions, limiting the accuracy of observations. Variable-pressure scanning electron microscopy (VP-SEM) offers an alternative approach, operating under low pressure rather than a high-vacuum environment, which can be advantageous for imaging live samples with minimal sample preparation. In this study, we applied VP-SEM to observe fiber cell initiation and early elongation in the conventional upland cotton cultivar UGA 230 at 0 and 1-day post-anthesis. Two SEM detectors, the ultra-variable-pressure detector and backscattered electrons, were used to capture detailed images. Optimal imaging conditions were identified with a 15 keV accelerating voltage and a 50 Pa pressure setting, enabling clear visualization of early fiber development without the need for extensive preparation. This VP-SEM protocol not only facilitates high-resolution imaging of cotton fibers at early developmental stages but also reduces time and expense, minimizing sample damage. Additionally, this optimized approach can be adapted for other fresh biological samples, making it a versatile tool for real-time imaging across various studies in plant biology and beyond.

## Introduction

Cotton plays an essential role in the global textile industry, with fiber quality and yield being critical for its economic success. The genus Gossypium, comprising over 50 species distributed across tropical and subtropical regions, demonstrates remarkable genetic diversity and morphological variation. Cotton fibers arise from single cells within embryo epidermal cells, but only about 25–30% of these cells ultimately develop into fibers (Haigler et al., 2012). The processes of fiber initiation and subsequent development are crucial determinants of cotton yield. Among the two types of fibers—lint fibers and fuzz fibers—lint fibers hold significantly greater economic value. Remarkably, no phenotypic differences can be observed between the two types of fibers during their initiation on the epidermal surface (Wu et al., 2006). Lint fibers begin their development at or just before the day of anthesis (-1 to 0 days post-anthesis, or DPA), elongating to lengths of 2–3.5 cm, whereas fuzz fibers initiate later, at 3–5 DPA, and reach much shorter lengths of 5–10 mm (Stewart, 1975). The diameter of mature fiber cells significantly influences fiber fineness, a key property for determining the mass per unit length of fiber. Fiber fineness is also impacted by the degree of secondary wall thickening. Among the various cotton species, Gossypium barbadense is notable for its smaller-diameter, tapered fibers, which are ideal for spinning fine, strong yarns used in high-quality fabrics (Bai and Scheffler, 2024). By contrast, Gossypium hirsutum produces a mixture of wide (hemisphere) and narrow (tapered) fibers, resulting in a broader range of applications in the textile industry (Stiff and Haigler, 2016; Graham et al., 2022).

Research into the early stages of cotton fiber development has traditionally relied on scanning electron microscopy (SEM). SEM offers highly detailed images of fiber structures, as exemplified by Stewart (1975) foundational work, which meticulously documented fiber development and the ovule’s surface structure during anthesis. However, SEM comes with significant limitations, including time-intensive sample preparation and challenges associated with imaging biological specimens (**Table 1**). Standard SEM operates in a high-vacuum environment to minimize ionization of gas molecules within the imaging chamber, which can distort electron beams and degrade image quality (Goldstein et al., 2017). Although this vacuum environment prevents potential sample ignition and eliminates static discharge from non-conductive samples, it also necessitates that specimens exhibit excellent electrical conductivity. Biological samples, which are typically poor conductors, must undergo extensive pre-imaging treatments, such as sputter coating with thin metallic films (e.g., gold, platinum, or palladium). Sputter coating involves generating an argon plasma that bombards a sample with metal particles, creating a conductive layer. While effective for SEM imaging, this process is highly invasive, as the low-pressure plasma environment, electrical currents, and metal bombardment can degrade biological samples. Additionally, chemical fixation and dehydration—steps necessary to prevent specimen shrinkage, off-gassing, and damage under low pressure—introduce artefacts, further complicating the interpretation of results. These challenges make SEM unsuitable for capturing real-time images of live or minimally prepared biological specimens. In addition to sputter coating, biological specimens typically undergo chemical fixation and dehydration to preserve their fine structure and prevent the boiling or off-gassing of water or other vapors during SEM imaging. However, these preparatory steps can cause shrinkage and other forms of structural damage that manifest as artefacts in the resulting images. Further damage may occur if any residual water or liquid remains in the specimen, as SEM is usually conducted under a hard vacuum that exacerbates these effects.

**Table 1:** Comparison of Variable Pressure Scanning Electron Microscopy (VP-SEM) and Traditional Scanning Electron Microscopy (SEM).

Feature	VP-SEM	SEM
Vacuum Level	Low to moderate (10–3,000 Pa)	High vacuum (~10^-^;^4^ Pa or lower)
Sample Preparation	Minimal; no coating required, can image hydrated/uncoated samples	Extensive; requires dehydration, fixation, and conductive sputter coating (e.g., gold, platinum)
Sample State	Hydrated, fresh, or living samples	Dry, fixed, non-conductive samples only
Resolution	Lower (due to electron scattering by gas molecules); ~1–10 nm range	Higher; sub-nanometer resolution possible (~0.4–5 nm)
Charge Neutralization	Gas molecules neutralize charge on non-conductive samples	Requires conductive coating to prevent charge buildup
Imaging Speed	Faster due to reduced preparation time	Slower due to extensive sample preparation
Dynamic Studies	Possible (e.g., hydration changes, pollen release)	Not feasible; samples must be static and dry
Applications in Plants	Stomata, trichomes, hydrated tissues, real-time processes	Fine structural details (e.g., cell walls, organelles after sectioning)
Artefacts	Fewer from dehydration/coating; some from gas interactions	More likely from drying, shrinkage, or coating
Equipment Cost	Higher; specialized system with variable pressure capabilities	Lower; standard SEM systems are more common
Ease of Use	Moderate; requires optimization of pressure and settings	Simpler once sample is prepared, but prep is labor-intensive

VP-SEM excels in plant science for its ability to image hydrated, uncoated samples quickly, making it ideal for studying natural plant surfaces or dynamic processes compared to SEM.

Given these challenges, there is a pressing need for imaging techniques that can provide high-resolution observations of cotton fiber development with minimal preparation and without compromising sample integrity. Such advancements could support efforts to improve cotton fiber development, focusing on producing high-quality lint fibers with desirable characteristics like narrow taped. To ensure the sustainability of cotton as a crop, it is essential to develop efficient methods for exploring fiber initiation and early elongation stages, with the goal of enhancing fiber yield and quality. Variable-pressure scanning electron microscopy (VP-SEM) offers a promising alternative to traditional SEM, especially for biological research (Nagaoka and Hirato, 2021). Unlike conventional SEM, VP-SEM operates under low-pressure conditions rather than a high vacuum. This distinction allows VP-SEM to image live or minimally prepared samples while preserving their natural structure and reducing preparation time. Research has utilized VP-SEM to examine leaf epidermal cells, trichomes, and stomatal guard cells in *Arabidopsis* and economically significant crops such as rice and cotton, providing detailed surface characterizations that support leaf viability assessments and taxonomic classifications (Talbot and White, 2013). VP-SEM has also been instrumental in studying the tracheids of opposite wood and various grades of compression wood in the corewood of *Pinus radiata* saplings. This research has revealed dimensional changes in longitudinal shrinkage and correlations with lignin and galactosyl residue content in the cell-wall matrix, which respond to environmental fluctuations (Zhang et al., 2018). Furthermore, VP-SEM enables real-time observation of pathogen colonization and biofilm formation on plant surfaces—critical for disease resistance research. It has been used to identify *Aspergillus fumigatus* biofilms, bacterial biofilms associated with beneficial nitrogen-fixing Rhizobia, and fungal hyphae and spore attachment on cereal crops. These insights contribute to the development of disease-resistant cultivars (Joubert, 2017; Joubert et al., 2017; Relucenti et al., 2021).

In our research, we employed VP-SEM to observe fiber initiation and early elongation in the upland cotton cultivar UGA 230. Observations were conducted at 0, 1 and 2 DPA, with two SEM detectors—an ultra-variable-pressure detector and a backscattered electron detector—used to capture detailed images of early fiber development. Through careful optimization, we determined that an accelerating voltage of 15 keV and a pressure setting of 50 Pa provided the best imaging conditions. These settings enabled high-resolution visualization of fiber cells without the need for extensive preparation, such as fixation, dehydration, or sputter coating. These advantages make VP-SEM a particularly valuable tool for studying fiber initiation and elongation in cotton, where early developmental stages play a critical role in determining fiber yield and quality. In addition to its application in cotton research, the VP-SEM protocol is versatile and can be adapted for other biological samples. This adaptability expands the potential uses of VP-SEM beyond plant biology, making it a powerful tool for a wide range of research areas that require minimally invasive imaging techniques.

## Materials and methods

### Plant growth and sample preparation

Plants of cotton UGA230, Tm-1, Gb3-79, KA3005, and UA222 varieties were grown in a greenhouse under controlled conditions. They were cultivated in 10-liter pots containing a sand and vermiculite mix, subjected to long-day conditions with 14 hours of light at 32°C and 10 hours of darkness at 25°C. Watering occurred twice weekly to ensure stable growth. Fresh ovaries were collected at 0, 1, and 2 days post-anthesis (DPA), starting on the day of flowering (0 DPA), as required.

To maintain ovule surface integrity and avoid fiber matting, we developed a specialized protocol for *in-situ* seed imaging within the locule. This method minimized handling and preserved the natural cotton fiber structure. Flower and early developing bolls were removed from the parent plant with 2–3 inches of peduncle attached to sustain hydration during processing. The severed peduncle ends were wrapped in moist paper towels to prevent drying, then placed, along with the towels, into either a 50 mL centrifuge tube or a plastic sandwich bag for transport. The flower and bolls were shipped overnight from Stoneville, MS, to Beltsville, MD, accompanied by ice packs to keep them cool. Upon arrival, they were promptly refrigerated at 4°C, remaining stable for same-day imaging. To prevent rapid ovary desiccation after boll removal, we ensured ovules remained attached to their hydration source by maintaining the vascular link between the ovules and the stem. Within the boll, each ovule is connected to a central stalk—an extension of the stem’s vascular tissue—allowing ovule surfaces to be exposed for imaging while preserving hydration through the intact vascular system.

**Figure 1:**
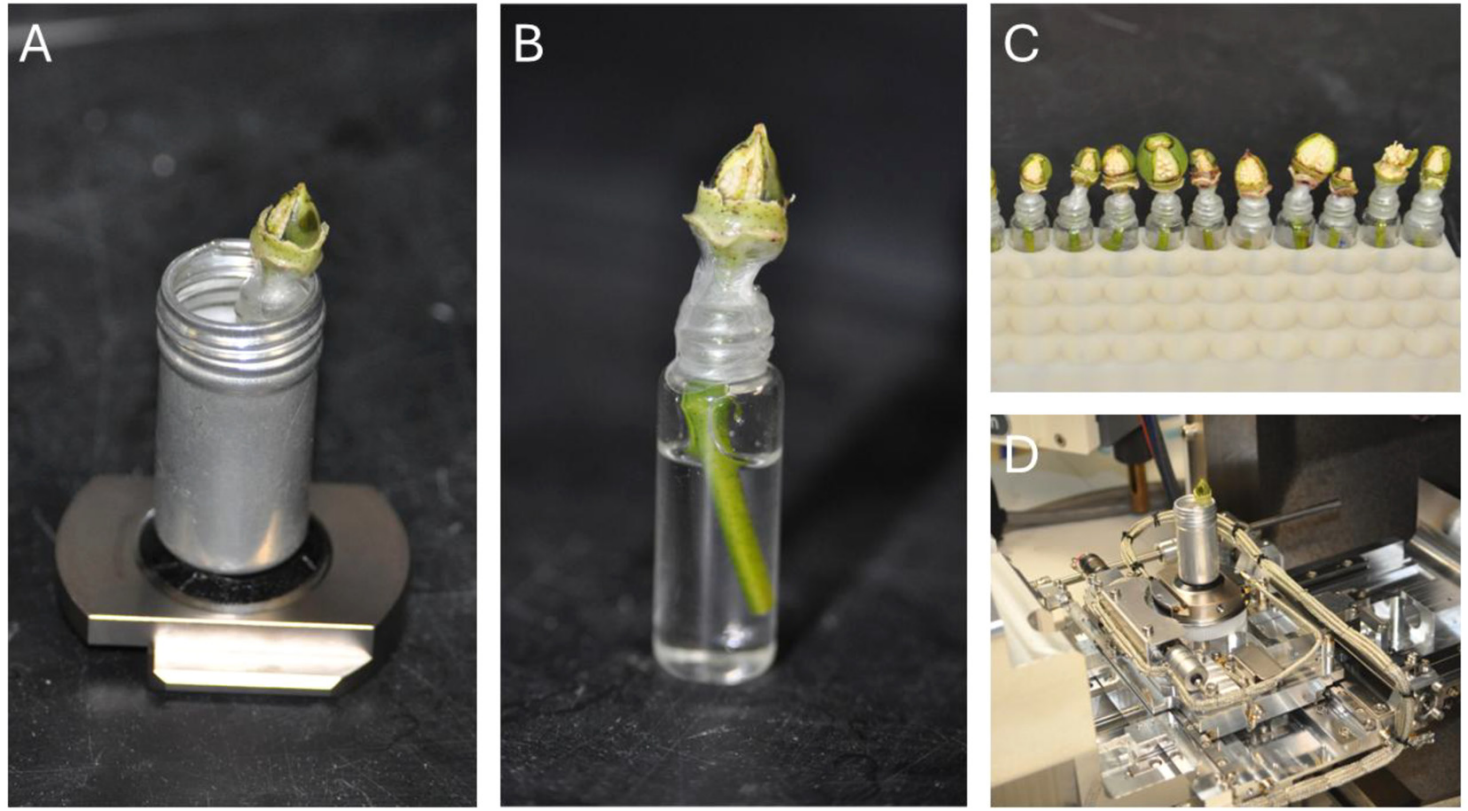
Preparation of fresh cotton samples for VP-SEM imaging. **(A)** Complete sample setup for VP-SEM, showcasing an aluminum canister secured to the SEM stage, containing the sample and a water source. **(B)** Magnified view of the sample, highlighting exposed seeds and an encapsulated water source, with samples maintained in a hydrate state using water. **(C)** Demonstration of rapid batch preparation of samples for imaging. **(D)** The aluminum canister functions as both a holder and a containment unit for the sample within the SEM.

To reveal the ovules, bolls were meticulously prepared using either a fine diamond-blade scalpel or a sharp steel blade to ensure precision and limit damage to fibers and adjacent tissues. Initially, the bracts and sepals were delicately detached from the boll. A triangular access window was then formed by executing a series of careful cuts into the boll’s outer layers. This involved trimming the terminal 1–2 mm of the boll tip to uncover the seed rows, followed by two longitudinal cuts along the sides of the targeted row and a shallow horizontal cut near the boll’s base. The excised tissue was lifted away with fine-tipped forceps, taking care to avoid disturbing the fibers or allowing sap to seep into the boll. When performed accurately, this technique exposed the seeds while preserving their hydration and structural integrity, with the vascular tissue linking the seeds to the stem left intact to sustain seed viability for imaging. This preparation enabled high-quality imaging of cotton seeds with minimal impact on fiber structures. **Figure 1** depicts the prepared 1-2 DPA bolls, highlighting the exposed seeds primed for *in-situ* imaging.

### Variable-pressure SEM imaging

All cotton seed samples were imaged using a Hitachi SU7000 Schottky-Emitter SEM using various VP-SEM detectors: secondary electron (SE) (PDBSE1, and PDBSE2), ultra-variable-pressure detector (UVD), alongside a light microscopy (LD) image. To preserve the delicate fine structure of the developing cotton fibers and mitigate commonly introduced artefacts caused by fixation, dehydration, sputter coating, *in-situ* imaging of the live tissue was performed using VP-SEM. VP-SEM is generally performed under conditions of low pressure, rather than hard vacuum, which has several distinct advantages (and disadvantages) for imaging live samples.

By introducing a small amount of gas into the vacuum chamber, secondary electrons emitted from the sample ionize nearby gas molecules close to the sample surface. Typically, this ionization prevents the secondary electrons from being detected for imaging, except with the lower detector positioned near the sample. However, this process also triggers the simultaneous emission of light (photons), which can be captured to produce an image. The SU7000 is equipped with a specialized Hitachi UVD designed to detect these emitted photons and generate a signal (Nagaoka and Hirato, 2021). Alternatively, the sample can be imaged using backscattered electrons (BSE), which possess sufficient energy to penetrate the ambient gas at low pressures. The presence of enough gas facilitates the dissipation of electrical charges from the sample surface, eliminating the need for sputter coating beyond a minimal pressure threshold. Furthermore, at sufficiently high pressure, the rate of liquid off-gassing is reduced, preventing the sample from bursting or rapidly drying out, thus allowing imaging in its natural hydrated state.

For optimal imaging with the UVD and BSE detectors, an accelerating voltage of 15 keV and a vacuum pressure of 50 Pa were found to yield the best results. As with traditional SEM, careful sample preparation remained essential to preserve cellular structure and fiber morphology (**Table 1**). To observe the developing fibers and cell surfaces, the seeds had to be exposed. However, completely removing the seeds from the boll often led to rapid desiccation and shriveling at 50 Pa, frequently damaging or matting the seed surfaces and fibers during extraction. Additionally, the bolls needed to remain intact for overnight shipment to the microscopy laboratory.

## Results and discussions

### Optimizing VP-SEM settings for imaging

Images of cotton fibers at initiation and early elongation were taken in VP mode at 50 Pa of vacuum pressure, using a 15kV accelerating voltage and high probe current, with 64 seconds for each integration. For each location, images from the lower SE detector (detector LD), PD-BSE detectors 1 (optimized for high signal sensitivity) and 2 (optimized for detecting compositional differences in the samples), and UVD were all taken simultaneously. Normally, to produce an image with SEM, a sample is irradiated with high energy electrons by using a focused electron beam. Some of these electrons cause ionization of atoms in the sample, which results in the emission of “secondary electrons” (SE) from the sample. Other electrons from the beam are deflected by the positively charged atomic nuclei in the sample; when the deflection angle approaches 180°, this is called “backscattering”, and the deflected electrons are called “Backscattered Electrons” (BSE). Different kinds of detectors can be used to measure the intensity of SE or BSE signals, or to measure other kinds of radiation (e.g. characteristic X-rays). To form an image, the beam is focused to a very fine point and moved (scanned) across the sample surface. At each location where the point is moved, the intensity of radiation emitted from the sample is measured by a detector and encoded as a numerical value. A raster of such values is created by moving the electron beam in a grid pattern across the sample, and by assigning a color to each value an image is generated. Normally, a weak signal is encoded as a darker color, and a stronger signal as a brighter color, which results in the typical grayscale/monochrome images associated with SEM.

The ability to image hydrated specimens means that for stationary biological samples (such as plants) *in-vivo* imaging is possible with VP-SEM. The most critical consideration for VP-SEM is gas pressure. If the pressure is high enough, static-electrical charges will not accumulate on the sample surface and photons will be generated in sufficient numbers to generate a clear image. Moreover, the rate of sample desiccation will also be low enough to allow for imaging of hydrated (or even live) samples. However, if the gas pressure is too high, both BSE and emitted photons will interact with the ambient gas, causing unacceptable degradation in image resolution. If the pressure is too low, the specimen will exhibit charging and the UVD signal will be too dim to form a clear image. At the proper gas pressure, there should be minimal specimen charging, strong signal with both the UVD and BSE detectors, and a low rate of specimen desiccation.

It is important to note that the stems of the fresh cotton bolls were kept immersed in water during this process to prevent the samples from drying out and shriveling. However, the need for constant hydration creates another problem for VP-SEM. The SU7000 is equipped with a variable-pressure module that enables vacuum pressure to be adjusted between 5-300 Pa. At 300 Pa, the boiling point of liquid water is -14.8°C, well below the ambient temperature inside the SEM. It was therefore necessary to create a means of hydrating the sample without exposing the source of water to low ambient pressure. Our solution was to encapsulate each boll with a water source attached to the stem of the boll. Once the seeds were exposed, each stem was trimmed with a sharp steel scalpel or micro-scissors and placed into a 5 mL glass vial filled with de-ionized water. The junction between the mouth of the vial and the stem of the boll was wrapped with parafilm and sealed with a liberal coating of clear nail polish, as shown in **Figure 1**. The nitrocellulose/acetate polymer was allowed to solidify fully. Once bonded to the parafilm, it was sufficiently rigid to maintain the internal pressure of the glass vial, thus preventing both fluid leakage and off-gassing from the encapsulated liquid water. For imaging, the glass vial would be wrapped in a folded lint-free tissue paper and placed into an aluminum canister affixed to the specimen stage with double-sided carbon tape. The purpose of the tissue paper was twofold: first, it serves as a packing to prevent the vial from moving or rotating inside of the aluminum canister, and second, in case the vial was to leak or break (this happened on one occasion), it would absorb the liquid to prevent any spillage inside of the SEM vacuum chamber. The aluminum canister thus acted as both a containment unit and a holster for the liquid-filled vial inside of the SEM (**Figure 1**).

### Early development of cotton fibers in the commercial UGA 230 line

**Figure 2:**
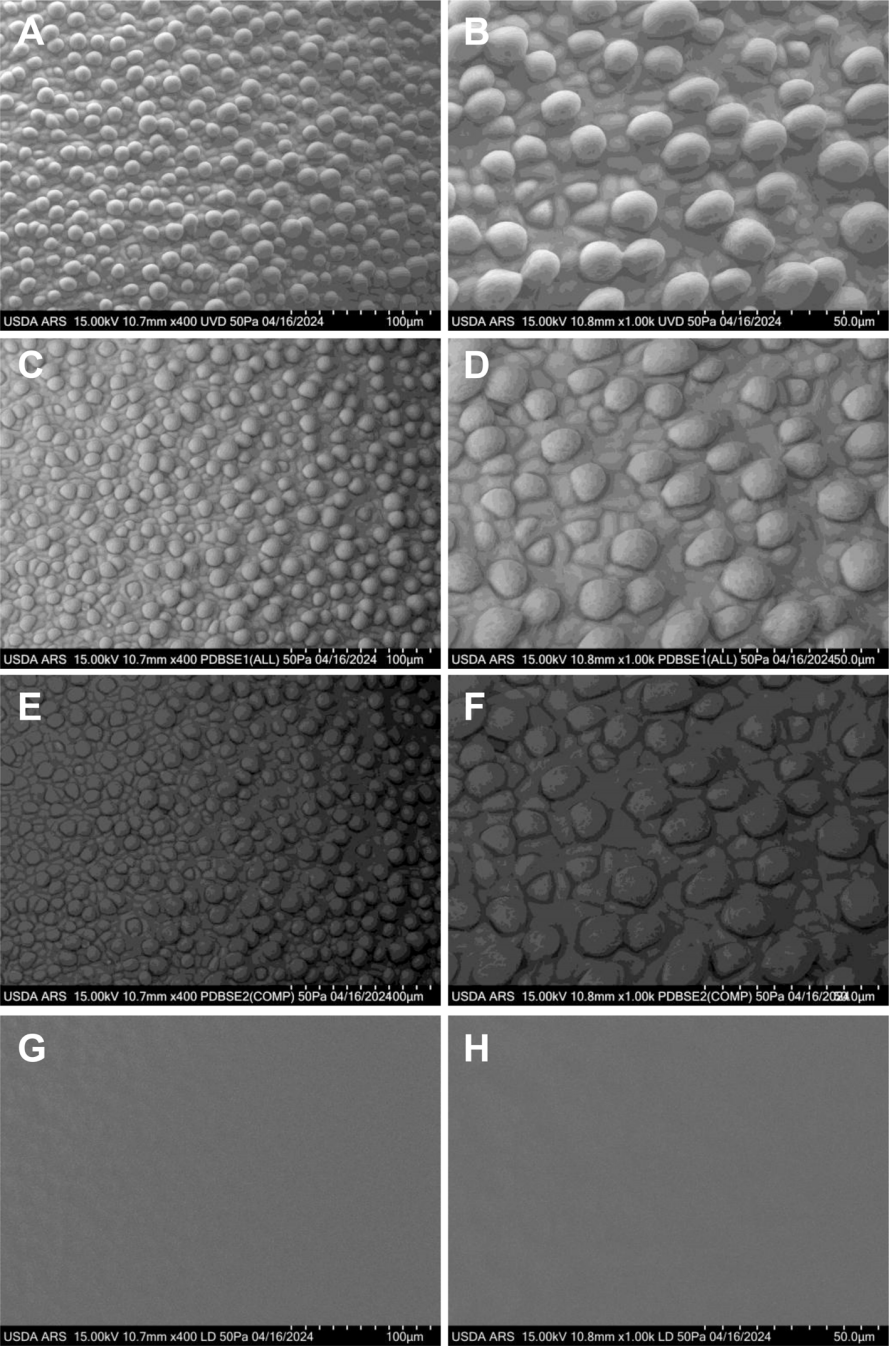
VP-SEM images of fresh cotton fiber initiation at 0 DPA in the UGA230 commercial cotton line, showcasing numerous spherical or hemispherical protrusions at the ovular midsection. **(A, B)** Captured with the UVD detector. **(C, D)** Captured with the PDBSE1 detector. **(E, F)** Captured with the PDBSE2 detector. **(G, H)** Captured with the LD detector. **(A, C, E, G)** Scale bar is 100 µm. **(B, D, F, H)** Scale bar is 50 µm. Images depict fiber initials as they round up and begin to expand. DPA, days post-anthesis.

UGA 230 is a conventional upland cotton cultivar developed and released by the University of Georgia Agricultural Experiment Station in 2009 (https://phytozome-next.jgi.doe.gov/sorghumpan/info/GhirsutumUGA230_v1_1). Renowned for its high yield potential, broad adaptability, and exceptional fiber quality, UGA 230 has made a significant impact on elite U.S. cotton germplasm. Its desirable traits—such as fiber length, strength, fineness, and uniformity—have led to its widespread use as a parental line in numerous public and private breeding programs, enhancing the development of superior cotton cultivars. The initiation of cotton fibers in the commercial UGA 230 cultivar was imaged at 0 days post-anthesis (DPA) using various VP-SEM detectors: PDBSE1, PDBSE2), UVD, alongside LD image (**Figure 2**). The UVD and PDBSE1 detectors provided superior resolution compared to PDBSE2, while the LD image exhibited the lowest quality. Before fiber initiation, the epidermal cell surfaces displayed a rectangular to irregular shape. However, as fiber development commenced, the differentiating cells became rounded and began to enlarge (**Figure 2**). Consistent with previous observations (Stewart, 1975; Li et al., 2009), fibers first emerged at the crest of the funiculus at anthesis and subsequently extended around the lateral circumference of the ovule. Morphological differentiation was marked by the rounding and protrusion of epidermal cells, which expanded diametrically before initiating elongation toward the micropylar end of the ovule. Initially, the protruding cells assumed a spherical shape above the epidermal layer, with a diameter approximately twice that of the surrounding nonexpanding cells.

**Figure 3:**
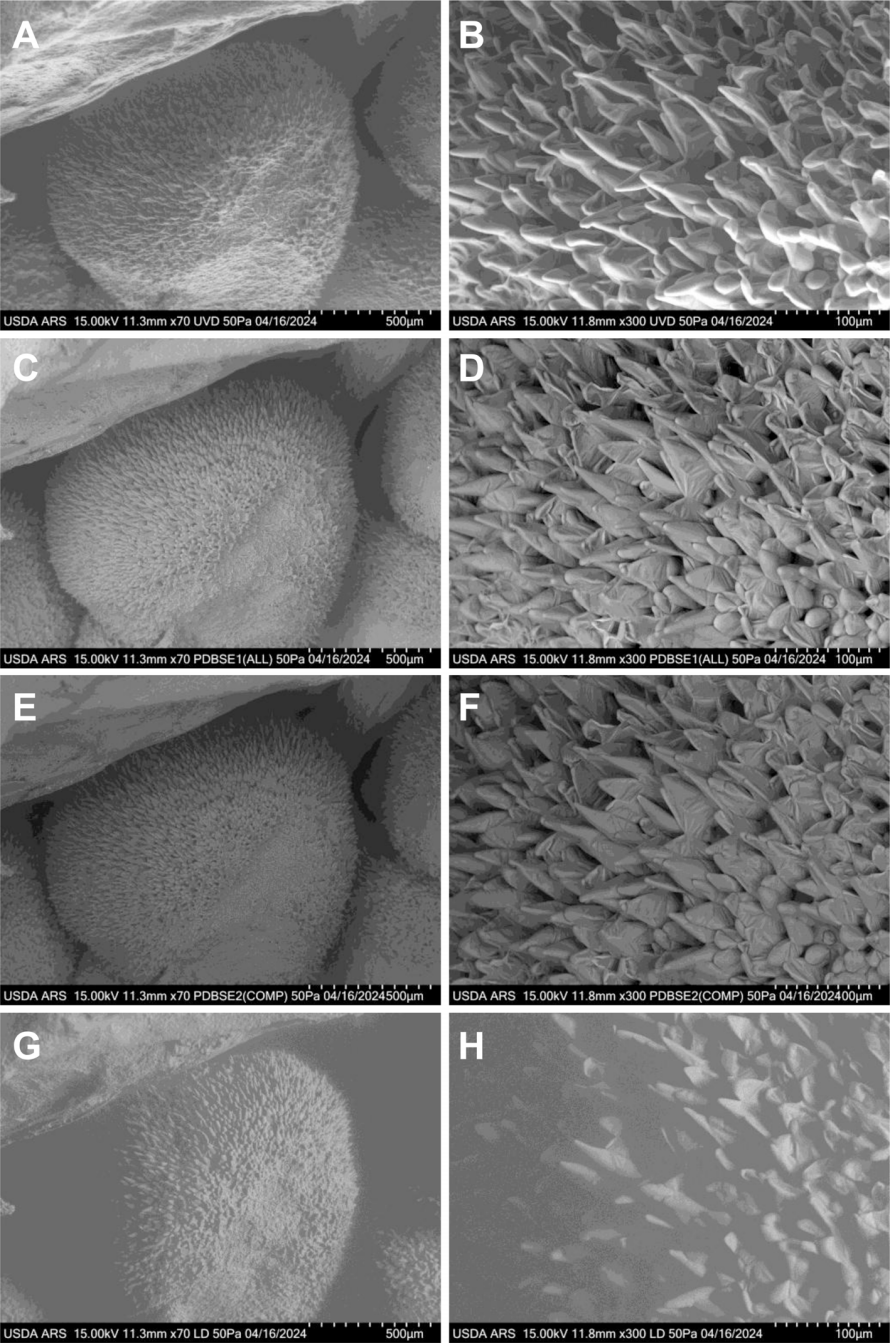
VP-SEM images capturing the early stages of fresh cotton fiber initiation at 1 DPA in the UGA230 commercial cotton line. **(A, B)** Imaged using the UVD detector. **(C, D)** Imaged using the PDBSE1 detector. **(E, F)** Imaged using the PDBSE2 detector. **(G, H)** Imaged using the LD detector. **(A, C, E, G)** Scale bar is 500 µm. **(B, D, F, H)** Scale bar is 100 µm. DPA, days post-anthesis.

Fiber elongation became increasingly evident during the first day post-anthesis (**Figure 3**). As the fibers lengthened, they began to adhere to adjacent fibers and, with continued growth, developed a spiral pattern. Over time, the initially blunt fiber tips became more tapered, reflecting further maturation. Fiber cell protrusions were observed across all tested cultivars, but the extent of protrusion and early elongation varied among materials. Interestingly, these differences in initial protrusion did not correlate with the ultimate lint percentage of the cultivars (Li et al., 2009). At 0 DPA, all samples exhibited significant numbers of fiber cell protrusions on the ovule surface, and by 1 DPA, these protrusions had elongated substantially. This consistent progression underscores the dynamic nature of early fiber development across different genetic backgrounds.

### VP-SEM imaging of additional cotton lines

**Figure 4:**
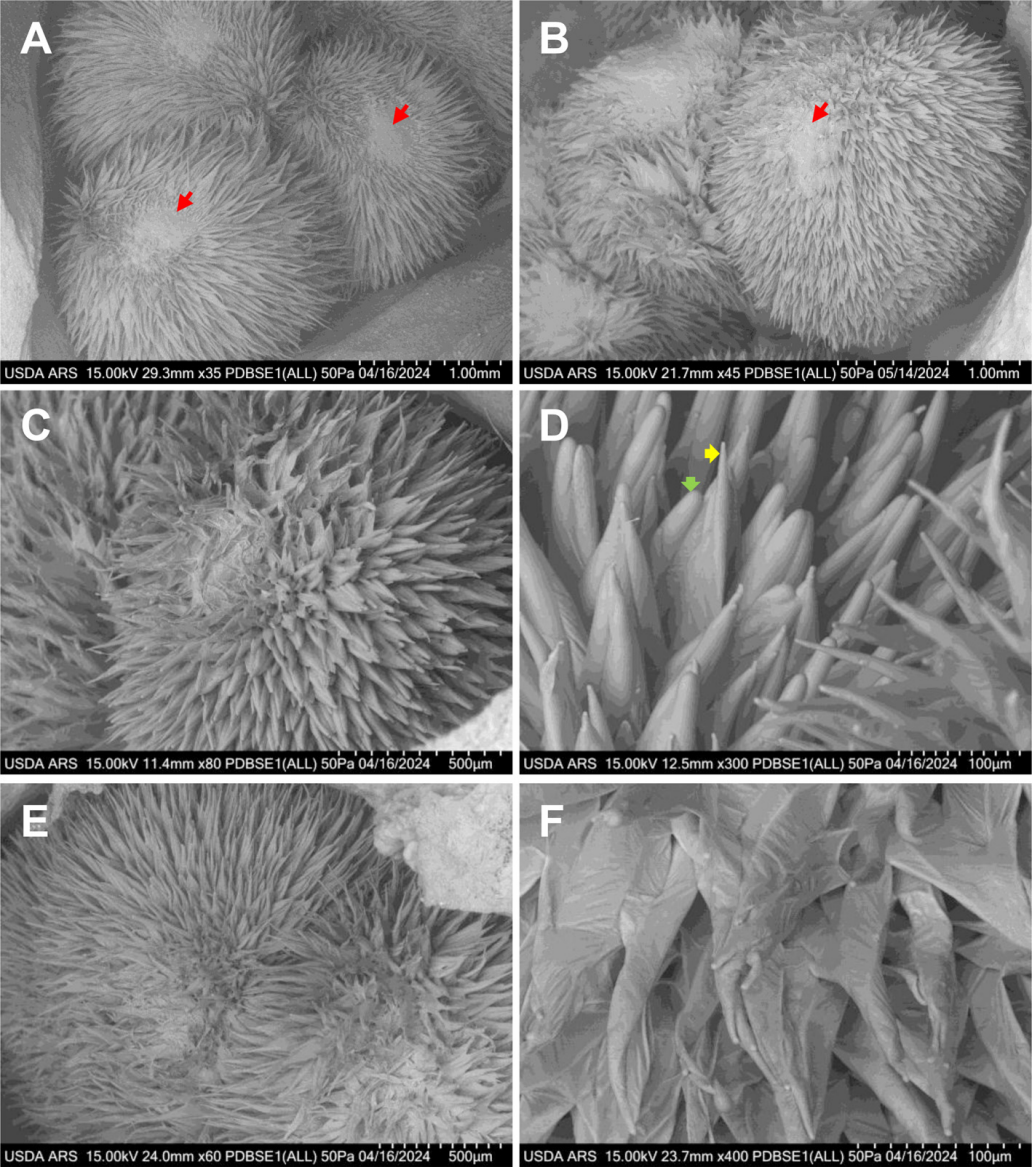
VP-SEM images illustrate fresh cotton fiber initiation and early elongation at different developmental stages across various cotton lines. **(A)** Gb3-79 ovary at 3 DPA, highlighting narrow tapered fiber cells. **(B)** Tm-1 ovary at 2 DPA, revealing a mix of narrow and wide tapered fiber cells. **(C, D)** KA3005 ovary at 2 DPA, displaying fibers grouped into clusters with two distinct blunt tip types: narrow (yellow arrow) and wide (green arrow); **(E, F)** UA222 ovary at 2 DPA, showing fibers elongating in a spiral formation. **(A, B)** Scale bar is 1 mm, with red arrows indicating the chalazal end. **(C, E)** Scale bar is 500 µm. **(D, F)** Scale bar is 100 µm. DPA, days post-anthesis.

*Gossypium barbadense* (Pima) cotton is characterized by narrow tapered fibers that are highly valued for spinning fine, strong yarns used in the most premium-quality fabrics. In contrast, *Gossypium hirsutum* (Upland) produces both wide hemisphere-shaped and narrow tapered fibers, which stabilize by the second day after anthesis and remain consistent through fiber maturity (Graham et al., 2022). The ratio of lint to fuzz fibers in *Gossypium hirsutum* plays a critical role in determining cotton yield. Tm-1, UA222 and KA3005 are Upland cotton varieties, whereas Gb3-79 is a Pima cotton line. Notably, both Tm-1 and Gb3-79 have been extensively deep-sequenced to serve as reference genomes for studying other cotton accessions. Pima cotton typically flowers later than Upland cotton, reflecting genetic differences in their reproductive timing. By approximately 2 DPA in Tm-1, UA222 and KA3005, and 3 DPA in Gb3-79, cotton fibers begin to segregate into small groups, develop tapered tips, and initiate spiral growth (**Figure 4**). Both Tm-1 and KA3005 exhibit two distinct types of blunt fiber tips at 2 DPA. As shown in **Figure 2**, KA3005 demonstrates narrow (yellow arrow) and wide (green arrow) fiber cell types, showcasing its structural variability.

In this study, VP-SEM offers an efficient approach for high-throughput imaging, enabling the examination of approximately 15 fresh cotton ovary samples per day. This method provides rapid estimation of lint fiber density, ovule and seed surface area, and the potential to determine fiber counts per ovule. These values can then be extrapolated to mature seeds, given that the total fiber number remains constant from ovule development to maturity. The percentage of narrow fiber cell types is a key determinant of cotton quality, and this imaging approach allows for the quantification of these traits. VP-SEM is particularly advantageous for monitoring rapid developmental changes in fiber tissues under varying environmental conditions, such as heat or drought stress—challenges that conventional methods struggle to address effectively. The technique allows high-throughput imaging of fresh or fresh-frozen reproductive plant samples collected at different DPA stages, making it especially valuable for breeding studies. Unlike conventional methods, VP-SEM eliminates the need for chemical processing, critical point drying, specialized cryo-accessories, temperature-controlled cold stages, or metal coating. This simplified protocol is user-friendly and is accessible to most microscopy laboratories. Additionally, machine-learning algorithms will be trained to calculate fiber cell types and fiber protrusion ratios, streamlining the selection process in agricultural breeding programs. By combining rapid imaging and automated analysis, this approach is poised to accelerate advancements in cotton breeding and improve adaptation to environmental stresses.

## Conclusions

Cotton remains an indispensable crop for the global textile industry, with its economic success dependent on continual improvements in fiber yield and quality. Traditional imaging methods, such as scanning electron microscopy (SEM), have provided valuable insights but are often constrained by labor-intensive sample preparation and risks of specimen damage. The VP-SEM protocol developed in this study offers several advantages over traditional SEM approaches. First, it minimizes sample preparation, reducing the time, expense, and potential artefacts associated with conventional methods. Second, it significantly reduces sample damage by eliminating the harsh conditions associated with sputter coating and high-vacuum imaging. Third, VP-SEM allows for real-time imaging of fresh samples, providing a unique opportunity to study dynamic biological processes as they occur. Through this advanced technique, optimal imaging conditions—15 keV accelerating voltage and 50 Pa pressure—have been identified, allowing for the precise visualization of early fiber initiation and elongation stages without the complications associated with traditional methods. By utilizing VP-SEM to study critical developmental processes, researchers can identify key factors influencing fiber growth, driving innovations that enhance both cotton yield and quality. The versatility and efficiency of VP-SEM extend well beyond cotton research, establishing it as an invaluable tool across multiple disciplines. In clinical research, recent studies have used VP-SEM to examine the marginal adaptation of novel bio-ceramic root repair materials, providing valuable insights into their clinical performance (DonFrancesco et al., 2024). Additionally, NASA is developing a VP-SEM designed to operate in extraterrestrial environments, obtaining CO_2_-rich atmospheres—such as those on Mars—to facilitate real-time imaging of geological and potential biological samples (https://techport.nasa.gov/projects/24815). As a streamlined, high-throughput imaging method, VP-SEM holds significant potential for advancing agricultural science and crop improvement, contributing to more sustainable and resilient farming practices in the face of global challenges.

## Data Availability

The original contributions presented in the study are included in the article/supplementary material. Further inquiries can be directed to the corresponding authors.

## Author's note

EEO/Non-Discrimination: USDA is an equal opportunity provider and employer.
